# Molecular detection of *Toxoplasma gondii* in *Cerdocyon thous* at southern Brazil

**DOI:** 10.29374/2527-2179.bjvm000725

**Published:** 2025-06-16

**Authors:** Julia Somavilla Lignon, Diego Moscarelli Pinto, Silvia Gonzalez Monteiro, Natália Soares Martins, Kauê Rodriguez Martins, Tamires Silva dos Santos, Giulia Ribeiro Meireles, Luíse Nunes Bonneau de Albuquerque, Rodrigo Casquero Cunha, Felipe Geraldo Pappen, Fábio Raphael Pascoti Bruhn

**Affiliations:** 1 Laboratório de Epidemiologia Veterinária, Departamento de Veterinária Preventiva, Universidade Federal de Pelotas, Pelotas, RS, Brazil; 2 Laboratório do Grupo de Estudos em Enfermidades Parasitárias, Departamento de Veterinária Preventiva, Universidade Federal de Pelotas, Pelotas, RS, Brazil; 3 Laboratório de Parasitologia Veterinária, Departamento de Microbiologia e Parasitologia, Universidade Federal de Santa Maria, Santa Maria, RS, Brazil; 4 Laboratório de Biologia Molecular Veterinária, Departamento de Veterinária Preventiva, Universidade Federal de Pelotas, Pelotas, RS, Brazil

**Keywords:** Apicomplexa, public health, toxoplasmosis, zoonosis, wild canids, Apicomplexa, saúde pública, toxoplasmose, zoonose, canídeos silvestres

## Abstract

Crab-eating fox (*Cerdocyon thous*) is widely distributed throughout South America, being the most common wild canid in Rio Grande do Sul, southern Brazil. It is considered a host for several parasites and contributes to the maintenance of its biological cycle due to its generalist and synanthropic habits. Due to the importance of the disease caused by *Toxoplasma gondii*, knowing its distribution in wild animals is essential to understand the transmission cycle of the protozoan. Therefore, the objective of the study was to report the molecular identification of *T. gondii* DNA in a sample of cardiac muscle tissue from *C. thous* in southern Brazil. For this purpose, a specimen of *C. thous*, found dead after being run over, was collected on the highways of Cerrito, Rio Grande do Sul, Brazil and sent to the laboratory for necropsy. Tissue fragments (spleen, liver, kidney, heart, lung, lymph nodes, bone marrow and blood) were collected and its genomic DNA was extracted. The samples were subjected to polymerase chain reaction (PCR) amplification using the 18S rRNA gene, and *T. gondii* DNA was amplified in cardiac muscle samples. The presence of the protozoan was confirmed by genetic sequencing. This study reports the molecular detection of *T. gondii* DNA in cardiac muscle of *C. thous* in southern Brazil, demonstrating the presence of the protozoan in the studied region. In addition, a new molecular sequence is being provided, contributing to the knowledge and epidemiology of the parasite.

## Resumo

O graxaim-do-mato (*Cerdocyon thous*) possui ampla distribuição pela América do Sul, sendo o canídeo silvestre mais comum no Rio Grande do Sul, sul do Brasil. É considerado hospedeiro de diversos parasitos e contribui para a manutenção do seu ciclo biológico devido aos seus hábitos generalistas e sinantrópicos. Devido à importância da doença causada pelo *Toxoplasma gondii*, conhecer sua distribuição em animais silvestres é essencial para a compreensão do ciclo de transmissão do protozoário. Portanto, o objetivo do estudo foi relatar a identificação molecular de DNA de *T. gondii* em uma amostra de tecido muscular cardíaco de *C. thous* no sul do Brasil. Para tanto, um espécime de *C. thous*, encontrado morto após atropelamento, foi coletado nas rodovias de Cerrito, Rio Grande do Sul, Brasil e enviado ao laboratório para necropsia. Fragmentos de tecido (baço, fígado, rim, coração, pulmão, linfonodos, medula óssea e sangue) foram coletados e seu DNA genômico foi extraído. As amostras foram submetidas à amplificação por reação em cadeia da polimerase (PCR) utilizando o gene 18S rRNA, e o DNA de *T. gondii* foi amplificado em amostras de músculo cardíaco. A presença do protozoário foi confirmada por sequenciamento genético. Este estudo relata a detecção molecular de DNA de *T. gondii* em músculo cardíaco de *C. thous* no sul do Brasil, demonstrando a presença do protozoário na região estudada. Além disso, uma nova sequência molecular está sendo fornecida, contribuindo para o conhecimento e a epidemiologia do parasito.

## Introduction

*Cerdocyon thous* Linnaeus, 1766 known as the crab-eating fox, is a wild canid widely distributed throughout South America, with significant occurrence in southern Brazil, especially in border regions with Uruguay and Argentina (Weber et al., 2020). It is the most common wild canid species in the state of Rio Grande do Sul (Dotto et al., 2001) and is frequently observed in rural and peri-urban areas. Their omnivorous habits, generalist behavior and high adaptability to anthropized environments favor their interaction with domestic and wild species, in addition to increasing exposure to infectious agents (Lignon et al., 2023; Weber et al., 2020).

Several studies indicate that wild animals, including carnivores such as C. thous, are susceptible to infection by *Toxoplasma gondii* Nicolle and Manceaux, 1908, an intracellular protozoan with a wide worldwide distribution (Torres-Castro et al., 2019). Toxoplasmosis is a relevant zoonosis, with the potential to affect a wide variety of warm-blooded hosts, including humans (Monteiro, 2017). Felines are the only definitive hosts of the parasite, responsible for the elimination of viable oocysts in the environment. Canines, although they do not play a direct role in the environmental dissemination of oocysts, can act as intermediate hosts through the ingestion of sporulated oocysts orally. In addition, they can become infected through the ingestion of infected prey with cysts in their muscles or congenital transmission of tachyzoites through the placenta (Monteiro, 2017; Silva et al., 2014).

In this context, as a top carnivore or mesopredator in some regions, *C. thous* can contribute significantly to the ecology of the protozoan as it can act as an indicator of environmental contamination and/or the circulation of *T. gondii* in wild food chains and can act as a sentinel, signaling potential risk of infection to other populations of animals (domestic and wild) and humans (Dakroub et al., 2023). Furthermore, although often asymptomatic, *T. gondii* infection can be associated with clinical effects in canids, including neurological, behavioral and reproductive alterations, which can have an ecological impact on vulnerable populations (Dubey, 2010). However, information on the epidemiology, occurrence and implications of toxoplasmosis in *C. thous* specially in southern Brazil is scarce, and most records are based on serological tests, without molecular confirmation (Gennari et al., 2004; Padilha et al., 2021).

Due to the importance of toxoplasmosis, knowing the distribution of *T. gondii* in wild animals is essential to understanding the transmission cycle of this protozoan. Therefore, the objective of the study was to report the molecular identification of *T. gondii* DNA in a sample of cardiac muscle tissue from *C. thous* in southern Brazil.

## Case description

An adult female Crab-eating fox (*C. thous*), found dead after being run over, was collected on the highways of Cerrito, RS, Brazil (31°43’48”S; 52°34’37”W). The animal had preserved and unexposed viscera, with an estimated death time of between one and seven hours, being collected and packed in a plastic bag, labeled with species, sex, date, city and the geographic coordinates where it was found and transported in an isothermal box with recyclable ice to the laboratory of the Parasitic Diseases Study Group, at the Veterinary Faculty of the Universidade Federal de Pelotas (UFPel), located in Capão do Leão, RS. In the laboratory, the animal was necropsied and tissue fragments such as spleen, liver, kidney, heart, lung, lymph nodes, bone marrow and blood were collected in duplicate, stored in sterile Eppendorf® microtubes and frozen at -20 °C ntil the Polymerase Chain Reaction (PCR) was performed. No macroscopic changes were observed.

The animal in the study is part of a scheduled research on trypanosomatid protozoa in wild animals in southern Brazil (Registration number Cobalto/UFPel 5604). The collection and transportation of roadkill wild animals were authorized by the Biodiversity Authorization and Information System of the Ministry of the Environment under registration 82632-3 based on Normative Instruction number 03/2014. This work was also approved by the Ethics Committee on the Use of Animals at UFPel (process number 23110.046990/2022-02).

Tissues DNA extractions were performed with commercial kits: PetNAD™ Nucleic Acid Co-Prep Kit for blood and ID Gene™ Spin Universal Extraction Kit for tissues, according to the manufacturer's instructions. The DNA samples were quantified in an ultraviolet light spectrophotometer (Thermo Scientific NanoDrop Lite Spectrophotometer, Waltham, Massachusetts, USA), to evaluate their quality by measuring their purity (260 nm/280 nm), using samples ranging from 1.8 to 2.2, and concentration in nanograms per microliter (ng/uL). Furthermore, electrophoresis was performed in 1% agarose gel to confirm the integrity of the extracted material.

PCR was performed using the following primers: F (5' CGCAAATTACCCAATCCTGA 3') and R (5' ATTTCTCATAAGGTGCAGGAG 3') amplifying a fragment of approximately 700bp of the 18S rRNA gene, according to Ferreira et al. (2018). In the reactions, 2.0 μL of DNA (50 ng/μL) and the mixture containing 2.0 μL of dNTP (2.5mM), 1.0 μL of each primer (10mM), 2.5μL of buffer solution (10X), 1.25 μL of MgCl2 (50 mM), 0.25 μL of Taq DNA polymerase (5U/μL) and 15 μL of ultrapure water were used, totaling 25 μL. Amplifications, in a conventional thermocycler, were: initial denaturation at 94 °C for 2 minutes, followed by 35 cycles at 95 °C for 45 seconds, 54 °C for 30 seconds, 72 °C for 60 seconds, final extension at 72 °C for 10 minutes, and 4 °C ∝ . As a positive control, *T. gondii* DNA was used, kindly provided by the Laboratory of Animal Protozoology of the Universidade Estadual de Londrina (UEL). Ultrapure water was used as a negative control. The amplified products were analyzed by electrophoresis in a 1.5% agarose gel, stained with ethidium bromide (0.5μg/mL) and visualized under ultraviolet light. A molecular weight standard of 100 bp was used (Ladder 100 bp 500 µl, Ludwig Biotechnology, Porto Alegre, Rio Grande do Sul, Brazil).

Amplicons were excised and purified using a Gel Purification Kit (Ludwig Biotechnology, Porto Alegre, Rio Grande do Sul, Brazil), according to the manufacturer's recommendations, and subjected to sequencing using the BigDye Terminator Cycle Sequencing Kit v3 .1 (Thermo Fisher, USA), using an ABI3500 genetic analyzer (Applied Biosystems, USA). Multiple sequence alignment was performed using the ClustalW method. Sequence similarity searches with sequences deposited in the National Center for Biotechnology Information (NCBI) database were performed using the BLAST tool (http://blast.ncbi.nlm.nih.gov/Blast.cgi). A maximum likelihood tree using Tamura 3-parameter model (Tamura, 1992) was constructed with the MEGA11 software (Tamura et al., 2021). Statistical analysis was performed using the bootstrap method on 1000 replications.

**Figure 1:**
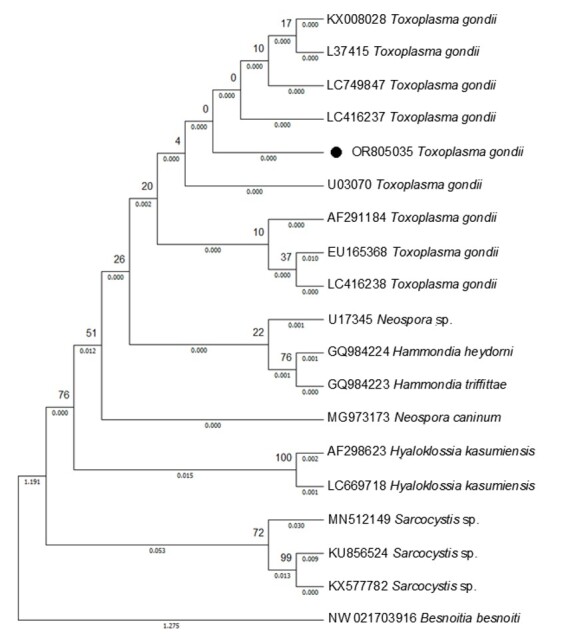
Phylogenetic tree for members of the phylum Apicomplexa based on 19 18S rRNA sequences and inferred by using the Maximum Likelihood method and Tamura 3-parameter model. GenBank accession numbers for all sequences are given in front of the taxon names. *Besnoitia besnoiti* was used as outgroup taxa.

The two *C. thous* heart muscle samples were positive for *T. gondii* in the PCR, amplifying products of approximately 700bp. The other samples (spleen, liver, kidney, lung, lymph nodes, bone marrow and blood) were negative. The presence of the protozoan was confirmed through genetic sequencing and the sequence obtained for the 18S rRNA gene from the heart sample was deposited in NCBI GenBank under accession number OR805035 (Figure 1). The 18S rRNA sequence obtained showed 99.85%-100% similarity with *T. gondii* (GenBank accession KX008028, L37415, LC749847, LC416237, U03070, AF291184, EU165368 and LC416238). Furthermore, the phylogenetic study based on the 18S rRNA gene sequences showed the positioning of both in the same clade.

## Discussion

Several studies have investigated the occurrence of *T. gondii* in wild carnivores in Brazil, including *C. thous*, with samples from roadkill, free-ranging or captive animals (Catenacci et al., 2010; Gennari et al., 2004; Marujo et al., 2017; Mattos et al., 2008; Nascimento et al., 2015; Richini-Pereira et al., 2016; Silva et al., 2014). In the south of the country, investigations were conducted in the states of Paraná (Gennari et al., 2004) and Rio Grande do Sul (Gennari et al., 2004; Padilha et al., 2021), focusing mainly on free-ranging animals. However, most of these studies used serological methods as a diagnostic tool. In contrast, the present study used the PCR technique for detection of protozoan DNA, an approach considered highly sensitive and specific for confirming infection (Dubey, 2010). Furthermore, the use of wild animals that have been victims of roadkill represents an ethical, practical and effective alternative for epidemiological studies, reducing the need for the use of live animals.

Previous serological studies have demonstrated a high exposure of Brazilian wild carnivores to *T. gondii* (Castiglioni et al., 2021; Gennari et al., 2004; Padilha et al., 2021); however, data regarding the molecular detection of the parasite are still limited, especially in the southern region of Brazil. In the country, only two studies reported the identification of *T. gondii* DNA in *C. thous*: Richini-Pereira et al. (2016), who detected the protozoan in the heart of an animal in São Paulo, and Almeida et al. (2017), who identified it in the brain of a specimen in Pernambuco. Thus, the present work contributes with new molecular sequences on *T. gondii* infection in *C. thous* in southern Brazil, expanding knowledge about the geographic distribution and tissue tropism of the parasite.

The detection of *T. gondii* DNA exclusively in the cardiac muscle in our study may be related to the protozoan's tropism for muscle tissues (in addition to the central nervous system and eyeball), especially during chronic infections, in which cysts containing bradyzoites tend to localize in tissues with low cellular turnover, such as the myocardium, favoring long-term persistence. The absence of DNA in the other analyzed tissues (spleen, liver, kidney, lung, lymph nodes, bone marrow, and blood) may reflect the absence of circulating parasites, which is consistent with the chronic phase of infection, when the proliferative forms of the parasite are no longer present in the bloodstream or in highly vascularized organs (Dubey, 1998, 2010; Tenter et al., 2000; Weiss & Kim, 2000). Moreover, previous studies conducted in Italy (Dakroub et al., 2023) and Serbia (Uzelac et al., 2019) showed that, similarly to our research, the protozoan's DNA has been predominantly identified in cardiac tissue samples compared to other tissues (e.g., brain and skeletal muscles) of wild mammals.

Although canids are considered accidental hosts of *T. gondii*, the impact of infections in these animals has not been fully elucidated to date. Infection in wild canids is often subclinical; however, Dubey et al. (2007) point out possible clinical repercussions associated with *T. gondii*, such as neurological, behavioral, or reproductive alterations in carnivores, in addition to indirect population consequences in natural environments under anthropogenic pressure. As the expansion of urbanization in natural areas is increasing contact between human populations and wild animals, wild carnivores may act as sentinel hosts or may be good indicators of the presence of the agent in the environment (Dubey & Beattie, 1988; Padilha et al., 2021). In this context, it is possible that the *T. gondii* infection reported in this research is related to the ongoing process of anthropization of the habitat and close contact with felines. Furthermore, we should highlight that this species commonly consumes prey highly exposed to the agent, such as rodents (Dubey, 2010), and therefore, transmission may also be related to their eating habits (Wolfe et al., 2001).

According to Catenacci et al. (2010), in wild environments, C. thous is hunted and sold for animal or human consumption and is often consumed rare. In southern Brazil, especially in the state of Rio Grande do Sul, sheep farming has great economic and cultural relevance, being one of the Brazilian states with the largest sheep herd. In this region, hunting crab-eating foxes is not only a cultural practice, but also a measure of control or retaliation, since the species is considered a predator of birds and sheep (Dotto et al., 2001). The presence of *T. gondii* cysts in the muscle tissues of wild animals represents a potential source of infection for both humans and domestic animals. In addition, hunters and their families can become infected during handling and evisceration of carcasses, due to direct exposure to contaminated tissues and fluids (Dubey, 1991). Conrady et al. (2022) described direct contact with the blood of infected animals and the dispersion of droplets resulting from the handling of game carcasses as a possible source of ocular toxoplasmosis.

In our study, no obvious macroscopic changes were observed in the organs evaluated during necropsy, including the heart. Histopathological examinations were also not performed, since the animal belonged to a scheduled study of trypanosomatid protozoa in wild animals in the region, becoming a limiting factor in our research that makes it impossible to evaluate microscopic changes associated with *T. gondii* infection. However, the molecular detection of *T. gondii* DNA in the cardiac muscle of *C. thous* has significant epidemiological importance, especially since it is a widely distributed wild animal that frequently inhabits areas at the interface between rural, urban, and wild environments, acting as a sentinel host for the environmental circulation of the parasite and contributing to the understanding of the dynamics of toxoplasmosis transmission in the region.

## Conclusion

This study reports the molecular detection of *T. gondii* DNA exclusively in the cardiac muscle of a *C. thous* specimen in southern Brazil, evidencing the circulation of the parasite in wild carnivore populations in the region. The finding contributes to the understanding of the transmission dynamics of the protozoan in environments shared by domestic and wild species. In addition, the molecular sequence generated in this study expands the genetic database of the parasite, aiding in future surveillance, diagnostic and comparative studies.
